# A brief feasibility report on an online psychosocial support intervention for adults with Li–Fraumeni syndrome

**DOI:** 10.3389/fpsyg.2025.1688050

**Published:** 2026-02-16

**Authors:** Senta Kiermeier, Christina Sauer, Madeleine Helaß, Juliane Nees, Myriam Keymling, Farina Silchmüller, Christina M. Dutzmann, Christian P. Kratz, Sarah Schott, Imad Maatouk

**Affiliations:** 1 Chair of Integrated Psychosomatic Medicine and Psychotherapy, Department of Internal Medicine II, University Hospital Würzburg, Würzburg, Germany; 2 University Cancer Center Frankfurt (UCT), University Hospital, Goethe University, Frankfurt, Germany; 3 Breast Unit, Sankt Elisabeth Hospital, Heidelberg, Germany; 4 German Cancer Research Center (DKFZ), Department of Radiology, Heidelberg, Germany; 5 Department of Pediatric Hematology and Oncology, Hannover Medical School, Hannover, Germany; 6 Department of Gynecology and Obstetrics, University Hospital Heidelberg, Heidelberg, Germany

**Keywords:** cancer, cancer predisposition syndrome, eHealth, Li–Fraumeni syndrome, psychooncology

## Abstract

**Introduction:**

Li–Fraumeni syndrome (LFS) is a rare autosomal dominant cancer predisposition syndrome characterized by a markedly elevated lifetime cancer risk. Despite the substantial psychological burden associated with LFS, tailored psychosocial interventions remain unavailable. Acceptance and commitment therapy (ACT) and cognitive behavioral therapy (CBT) are evidence-based approaches widely applied in psycho-oncological care; however, no intervention has been specifically adapted for individuals with LFS. Consequently, we developed OnLiFe, an online self-management program designed to meet the unique needs of this population and investigated its acceptability and practicality in a small adult cohort.

**Methods:**

OnLiFe comprises six modules focusing on psychoeducation about emotions, mindfulness practices, and resource activation. Acceptability and practicality were assessed in terms of comprehension and satisfaction with content, usefulness, simplicity, and integrability for each module and content element. Qualitative feedback was collected through open-ended questions and analyzed using qualitative content analysis.

**Results:**

Nine female participants (mean age 46.0 ± 7.1 years) enrolled, of whom four completed all modules. Participant satisfaction with OnLiFe was rated moderate to high, while perceived helpfulness of the content showed relatively lower ratings. Qualitative data indicated that the intervention was perceived as too lengthy and that participants' busy schedules limited full engagement.

**Conclusion:**

Given the distinct challenges of LFS, tailored psychosocial support is required. Despite careful theoretical considerations, OnLiFe showed mixed results regarding acceptability and practicality. Addressing time constraints will be essential, and future studies are warranted to optimize and determine the efficacy of an adapted version of OnLiFe.

## 1 Introduction

Li–Fraumeni syndrome (LFS) is a rare, inherited cancer syndrome that predisposes a very high lifetime risk of developing multiple malignancies from early childhood (Kratz et al., 2022). It is caused by underlying alterations in the tumor suppressor gene *TP53*. Compared with the general population, individuals with LFS experience a shortened life span due to their elevated cancer risk. Therefore, individuals are advised to adhere to an intense cancer surveillance protocol aimed at early detection and interception of cancers (Kratz et al., 2017; Villani et al., 2011). LFS is considered a rare disease, with an estimated 20,000 individuals affected in Germany (Dutzmann et al., 2019; Penkert et al., 2022).

Individuals with LFS have dynamic psychosocial support needs due to intensive cancer screening and screening-associated anxiety, personal and family cancer diagnoses and bereavement, and difficulty finding experts to manage LFS (Kiermeier et al., 2025, 2024). The use of online technologies appears promising, as they can be accessed irrespective of location or time, are readily available to users, and can be personalized according to shifting needs. To address psychosocial needs for cancer patients or survivors, the use of online tools (e.g., websites or smartphone apps) has been gaining more and more interest in the past decade (McAlpine et al., 2015; Seiler et al., 2017). The use of eHealth in cancer care has expanded considerably in recent years. This is exemplified by digital health applications, which have been prescribable in Germany since 2020, including some cancer-specific apps (https://diga.bfarm.de/de/verzeichnis). Furthermore, eHealth is also increasingly being integrated and utilized within cancer predisposition syndrome–specific patient populations, for example, an online decision support tool for couples affected by *BRCA1/2* (a more common CPS than LFS), which has been pilot tested and revealed positive usability ratings (Reumkens et al., 2019).

Cancer diagnosis, treatment, and survivorship often denote a psychosocial burden that may result in cancer worry, distress, depression, anxiety, and impairment of quality of life (Riba et al., 2019; Vehling et al., 2022). For LFS, the process of genetic counseling and cancer surveillance often represents additional psychosocial challenges (Bancroft et al., 2020; Eijzenga et al., 2014). Consequently, individuals with LFS and their (often also affected) family members are at even higher risk for psychological symptoms even before an actual cancer diagnosis (Forbes Shepherd et al., 2018; Kiermeier et al., 2024; Lammens et al., 2011). Extensive symptoms of fear of progression (FoP) have been previously described for adult individuals with LFS (Kiermeier et al., 2024).

Cognitive behavioral therapy (CBT), a form of psychotherapy that focuses on identifying and changing negative thought patterns and behaviors, is a reliable approach in coping with these and other cancer-related burdens (e.g., fatigue) (Johnson et al., 2016; Xia et al., 2024; Zhang A. et al., 2022; Zhang L. et al., 2022). Evidence also suggests that the “third wave” psychotherapeutic approach of acceptance and commitment therapy (ACT) is efficacious for cancer patients and survivors regarding quality of life, depression, anxiety, and distress (Sauer et al., 2024). ACT aims to enable a productive and fulfilling life (e.g., despite feelings of anxiety due to a chronic disease). ACT uses the hexaflex model as a theoretical framework that includes six core processes: cognitive defusion (creating distance to thoughts), experimental acceptance, self-as-context, values, committed actions, and present-moment awareness (see Supplementary material A). According to ACT, these processes interact with each other and overall foster psychological flexibility. Psychological flexibility facilitates coping and overall psychological wellbeing (Sauer and Weißflog, 2022). Currently, no study has specifically examined the use of CBT and ACT for individuals with LFS (Sauer et al., 2024).

Based on evidence supporting the effectiveness of CBT and ACT for cancer patients and survivors, we developed an online psychological intervention designed for individuals with LFS. Because LFS is a rare disease, a digital intervention was developed to reach individuals in geographically diverse locations in Germany. Moreover, digital interventions can be used asynchronously, which may reduce the burden of participation among an already burdened population (e.g., intensive LFS management, distress).

As this is the first online psychosocial intervention specifically designed for individuals with LFS, first, we aimed first to report the development of the intervention and second to investigate its acceptability (participants' satisfaction, intent to continue to use, and perceived appropriateness) and practicality (participants' perceived positive/negative aspects, and the ability of participants and the study team to carry out the intervention) in a small adult LFS cohort.

## 2 Material and methods

### 2.1 Intervention

Based on CBT and ACT as theoretical frameworks, the OnLiFe (online psychosocial support program for individuals with Li–Fraumeni syndrome) intervention was comprised of six modules. Each module opened and closed with a guided mindfulness exercise delivered through audio recording, followed by a psychoeducational section demonstrated with images and accompanied by free-text fields inviting participants to reflect and respond to guiding questions. Midway through and at the end of each module, a “section milestone” summarized key concepts covered to that point. Each module concluded with a “Suggestion for the Coming Week,” offering practical guidance on how to apply the content in daily life. OnLiFe was designed as a self-management intervention. However, participants could contact the study team through chat or e-mail in case of questions, with responses provided within 48 h on weekdays. The estimated average completion time per module was 20–30 min, and participants were asked to finish each module within 4–7 days; automated e-mail reminders were sent if deadlines were exceeded. The intervention was created and delivered using the secure, web-based Minddistrict^®^ platform.

The following outlines adaptations of ACT and CBT content for the context of LFS. To promote cognitive defusion, psychoeducation and exercises were provided (e.g., reframing “I am sick” to “I have the thought that I am sick”). Experiential acceptance was introduced through psychoeducation, a thought-control exercise, and the “fate diagram,” which prompted reflection on controllable versus uncontrollable aspects of life; classic ACT metaphors reinforced this concept. Values work included reflecting on physical, behavioral, and cognitive changes since diagnosis, identifying neglected life areas (e.g., hobbies), and re-considering the impact LFS has in daily life. Participants also ranked a provided list of values according to their personal importance. Present-moment awareness was addressed through mindfulness audio exercises (e.g., mindful breathing) in each module, with the first module including psychoeducation on mindfulness.

**Figure 1:**
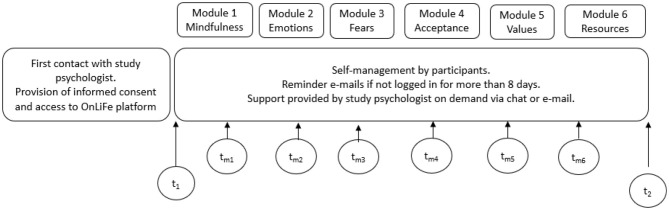
Study design.

The mainly CBT-oriented parts of OnLiFe included resource-oriented exercises (e.g., assembling a “tool-box” of helpful strategies, reflecting on one's social network, support opportunities, and positive activities). A validated CBT-based approach was used in this study for dealing with FoP in cancer patients (Herschbach and Dinkel, 2014). The quality of the intervention was confirmed by the fact that the authors are trained in these therapeutic approaches: CBT: MH, CHS, SK (in training); ACT: CHS. As shown in Figure 1, the OnLiFe feasibility study was conducted with a pre (*t*_1_) and post (*t*_2_) questionnaire on psychosocial outcomes, together with six additional evaluation questionnaires (*t*__*m*_1 − 6_) after each module.

### 2.2 Data collection and material

**Figure 2:**
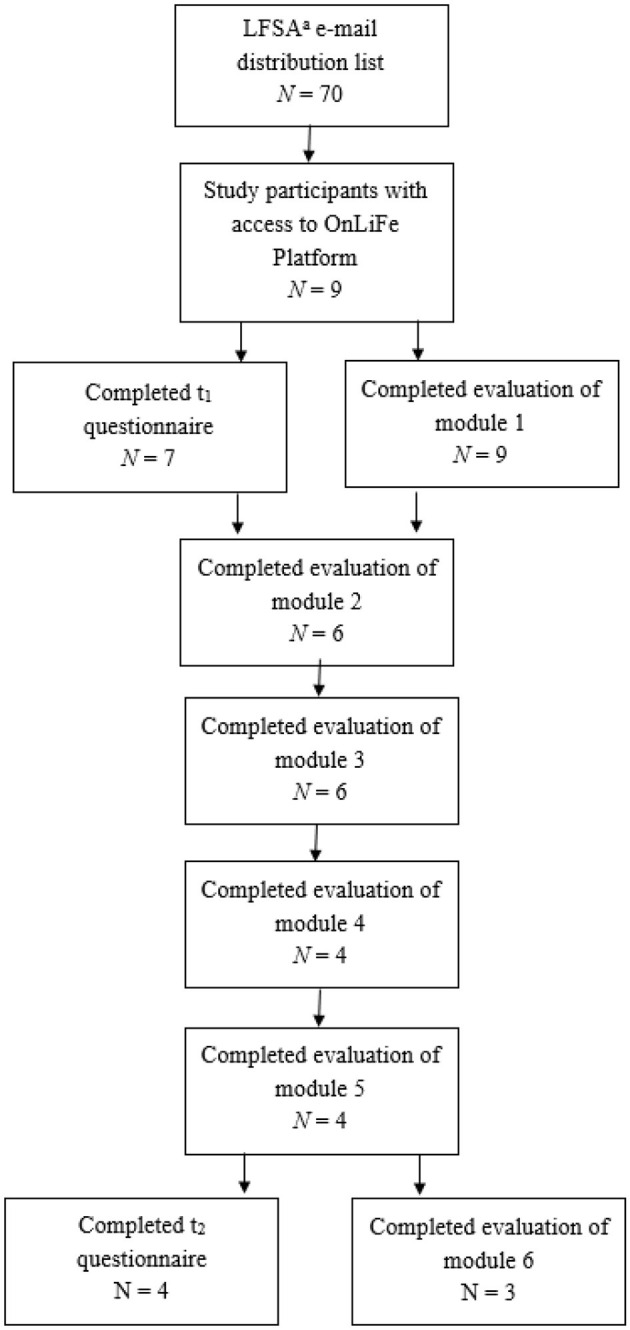
Flow diagram of study population. ^a^Li–Fraumeni syndrome Association, Germany.

Adults (≥18 years) with a confirmed LFS diagnosis were eligible. The intervention was conducted in collaboration with the Li–Fraumeni Syndrome Association Germany (July–October 2023). Recruitment occurred through the association's mailing list (≈70 members) and through a presentation at an LFS information event at Heidelberg University Clinic (February 2023). After providing written informed consent, participants received pseudonymized e-mail access to the online intervention for independent use. A flowchart is shown in Figure 2. This study is part of a larger-scale study on the psychosocial needs of individuals with LFS (Kiermeier et al., 2025, 2024).

### 2.3 Measures and data analysis

The following self-designed measures were used to examine the acceptability and practicality of OnLiFe. Each module ended with a short questionnaire (*t*__*m*_1 − 6_) assessing satisfaction, comprehensibility, and motivation on a 6-point Likert scale (0–5). At the final module (*t*_2_), participants rated each content and design element (e.g., mindfulness exercises, text, videos, images, self-reflection questions) using the German school grading scale (1 = best to 6 = worst). Additional items at *t*_2_ included usability, duration, and comprehensibility, and recorded an average module completion time (10–20 min to >90 min). All questionnaires were optional, resulting in missing data (not imputed; see Figure 2). Open-ended questions explored reasons for dropout, satisfaction, challenges, suggestions for improvement, and positive aspects (e.g., “What aspects of the program did you find particularly positive?”). The intervention's final question—“What do you want to remember from the past weeks? What is important to you after the end of OnLiFe?”—was also included as qualitative data. In addition, basic demographic and LFS-related variables were evaluated (e.g., age, relationship status, and known family members with LFS). The descriptive statistics, including absolute and relative frequencies, means, and standard deviations for quantitative measures, were also calculated. For qualitative data, a qualitative content analysis was carried out (Mayring, 2017). In line with this approach, inductive categories derived from the open-ended questions were established, and participant responses were assigned to these categories, with revisions made as necessary.

## 3 Results

### 3.1 Participants

Nine women registered for this study through e-mail and had access to the OnLiFe platform (response rate 12.9%). The demographic data are available for seven participants and are shown in Table 1. Age of participants ranged from 36 to 54 years (*mean* = 46, SD = 7.14). Over the course of the intervention, five participants dropped out (completer rate: 44.4%). Some reasons are available, e.g., one participant stated via e-mail, “I'm currently very stressed by the program.”

**Table 1:** Sample characteristics (*N* = 7).

**Sample characteristics**	***N***	**%**
**Sex**		
Female	7	100
**Marital status**		
Single	1	14.3
Married/relationship	6	85.7
**Highest school-leaving qualification**		
Basic schooling/10th grade	2	28.6
12th/13th grade	3	42.8
Bachelor's, Master's, or doctoral degree	2	28.6
**Occupational status**		
Employee	6	85.7
Children		
Yes	4	57.1
**Children with a known LFS diagnosis**		
Yes	2	28.6
**Known number of family members with LFS**		
None	2	28.6
One or two	3	42.9
More than six persons	2	28.6
**Number of family members with cancer diagnoses**		
One to three persons	3	42.9
Five to six persons	2	28.6
More than six persons	2	28.6

LFS, Li–Fraumeni syndrome.

### 3.2 Quantitative results

The results of the module evaluations (*t*__*m*_1 − 6_) are shown in Table 2. Participants were asked to rate the overall intervention from 1 (“no/not at all”) to 5 (“very much”) for several categories: overall understanding of content (*mean* = 2.25, SD = 0.96), content satisfaction (*mean* = 4.00, SD= 0.0), design of OnLiFe (*mean* = 4.25, SD = 0.96), usefulness of FAQ-content (*mean* = 2.25, SD = 0.96), met expectations (*mea*n = 4.00, SD, = 0.82), usefulness for everyday life (*mean* = 4.00, SD = 0.82), simplicity of use (*mean* = 4.25, SD = 0.96), integrability in everyday life (*mean* = 4.00, SD = 1.16), and satisfaction with technology (*mean* = 1.50, SD = 0.56).

**Table 2:** Results of descriptive analysis of module analysis (*t*__*m*_1 − 6_).

**Items of module evaluation**	**Module 1 (*N* = 9)**	**Module 2 (*N* = 6)**	**Module 3 (*N* = 6)**	**Module 4 (*N* = 4)**	**Module 5 (*N* = 4)**	**Module 6 (*N* = 3)**	**FAQ^a^ (*N* = 2)**
	**Mean (SD)**						
Overall satisfaction	4.33 (0.87)	4.17 (0.75)	3.67 (1.37)	4.50 (0.56)	4.00 (0.82)	4.33 (0.58)	3.50 (0.71)
Understanding of content	4.56 (0.53)	4.33 (0.52)	4.17 (0.75)	4.50 (0.58)	4.50 (0.58)	5.00 (0.00)	4.50 (0.71)
Knowledge gain	3.33 (1.0)	3.50 (0.84)	3.67 (0.52)	4.25 (0.96)	3.50 (0.58)	4.00 (1.0)	2.00 (0.00)
Helpfulness of content	4.11 (1.27)	3.83 (0.75)	3.67 (1.37)	4.25 (0.96)	4.25 (0.50)	3.67 (1.53)	3.5 (0.71)
Motivation to continue OnLiFe	4.67 (0.71)	4.33 (0.82)	4.00 (0.89)	4.25 (1.5)	4.25 (1.5)	4.67 (0.58)	3.5 (0.71)

Range: 1 = no/not at all to 5 = very much. ^a^Section with “Frequently Asked Questions”; Each module focuses on a specific content: Module 1 = mindfulness, Module 2 = emotions, Module 3 = fears, Module 4 = acceptance, Module 5 = values, Module 6 = resources.

The mean grades (1 = best, 6 = worst) were 2.75 (SD = 0.96) for videos, 1.50 (SD = 0.58) for texts, 2.00 (SD = 1.16) for pictures, 1.25 (SD = 0.50) for mindfulness practices, 1.75 (SD = 0.50) for self-reflective practices, and 2.00 (SD = 0.82) for homework.

The reported time to complete each module ranged from “10–20 min” (*N* = 1), “21–30 min” (*N* = 2), to “51–60 min” (*N* = 1) and was rated as “exactly right” by all four completers. The online format was rated by all as “just as good as on-site/presence”. Three completers were “absolutely sure” that they would take part in an online intervention for LFS again, and one person rated “maybe/don't know”.

### 3.3 Qualitative results

In the following section, we present the results of the qualitative content analysis of the open-ended questions. Categories were generated inductively from the material and are shown in more detail in the Supplementary material B.

#### 3.3.1 Positive feedback

The main category “positive feedback” comprised five subcategories: mindfulness exercises, other exercises, psychoeducation, videos, and general positive feedback. Mindfulness exercises were frequently appreciated (e.g., “*I love such instructions; they help me overcome my inner inertia,” “The mindfulness exercises have been very helpful to me”*). In the “other exercises” category, participants highlighted exercises aiming at the acceptance aspect of the hexaflex, the CBT exercise of confronting oneself with their fears, and reflecting on “personal values” (e.g., “*What I particularly liked were the reflections on personal values. The examples were also good”*). Psychoeducational content on emotions such as anxiety and on the scientifically validated benefits of mindfulness was also well received. Three participants provided explicitly positive feedback on the videos (e.g., “*The video sequences should be retained*”).

#### 3.3.2 Negative feedback and process difficulties

The “negative feedback” category comprised three subcategories: time, technology, and content. Regarding time, some participants felt stressed by the intervention's length (e.g., “*I am stressed out about the time I need*”) or reported difficulty in finding quiet time for OnLiFe, with one noting that two breathing exercises were too time-consuming. Technological issues included difficulties navigating the platform, criticism of video implementation, a too-quiet audio voice, and the inability to access the FAQ section. Content-related feedback ranged from perceptions that the modules “*did not promote depth*” to descriptions of content as very complex. One participant expressed reluctance to engage with the LFS topic, and another reported experiencing a “*major setback*” due to a failed surgery during the study period.

#### 3.3.3 Change requests

The final main category comprised two subcategories: changes to exercises and to the content design. Regarding exercises, participants requested more practical, varied, and smaller tasks, accompanied by concrete examples (e.g., “*I would prefer more small and varied exercises*”). Suggestions for content design included providing greater depth for familiar topics, offering options to tailor modules to individual preferences (e.g., addressing or omitting the topic of family planning), and adding support for managing worries, fears, and homework. One participant proposed follow-up and more detailed discussion of previous module homework (“*It would have been nice to follow up on the homework from the previous module and potentially address it in more detail*”).

## 4 Discussion

The study aims first to provide information on how to develop an online support intervention for adults with LFS and second to investigate the acceptability (participants' satisfaction, intent to continue to use, and perceived appropriateness) and practicality (participants' perceived positive/negative aspects and the ability of participants and the study team to carry out the intervention). Based on the findings from a small sample of adults with LFS, it appears that OnLiFe participants reported moderate to high satisfaction with OnLiFe, high understanding of the content, and completers had high motivation to continue. Qualitative feedback revealed contentment with mindfulness exercises and psychoeducation. However, participants also reported low to moderate helpfulness of content and knowledge gain. Qualitative data also disclosed time constraints and change requests for the program design (e.g., more exercises). Additionally, out of nine initial participants, only four completed the program. Questionnaires were partly omitted, leading to missing data.

### 4.1 Intervention design

As therapist-guided eHealth interventions appear to be more efficacious than self-guided formats, this should be considered in the design of future LFS online interventions (Seiler et al., 2017). An exposure to the existential LFS-related fears (sickness, death, or loss of family members) may be too complex to address at a self-management level and may require more guided support, as highlighted in quantitative feedback. These qualitative results, along with previous research (Kiermeier et al., 2024; Nees et al., 2022), demonstrate that individuals with LFS can be in very different health situations (e.g., as the underlying *TP53* variants differ in intensity (Kratz et al., 2021), life stages (e.g., having children), and information needs (e.g., varying experience due to different times since initial diagnosis). Previous research shows that family planning is complex and emotionally challenging for individuals with LFS (Kelly, 2009). This was reflected in the study cohort, where one participant requested more information on pre-implantation genetic diagnostics, while another declined receiving any information, considering the matter resolved. The results suggest participants prefer a less rigid intervention structure, favoring flexibility to select topics most relevant to them. This indicates that a uniform program may not adequately meet the needs of all individuals with LFS.

### 4.2 Recruitment

For eHealth interventions, low response rates are common (Grapp et al., 2022). Despite recruitment being open for all genders and ages, exclusively middle-aged women took interest in this study. This aligns with previous findings, as women often assume the role of “health leaders” in LFS families, which comes with LFS-related duties and responsibilities (Pantaleao et al., 2020). In addition to the elevated breast cancer risk and concerns related to family planning, women may have a greater need for additional psychosocial support. However, a previous study did not find significant differences in psychosocial burdens between male and female participants in the previous study (Kiermeier et al., 2024). When recruiting for future LFS-focused psychosocial interventions, specific strategies may be considered to better engage men, such as the design of recruitment materials (Sörensen et al., 2018). In this study, approximately 70 individuals with LFS were contacted, of whom 9 ultimately participated, yielding a response rate of 12.9% and a completer rate of 44.4%. These findings are consistent with the generally higher dropout rates reported in online interventions compared with other studies reported in the literature (Hernandez-Rodriguez et al., 2022). This study therefore recommends planning for a high recruitment effort in online intervention studies for individuals with LFS. In this small cohort, one person reported increased anxiety during the module evaluation due to a failed medical procedure. For studies involving individuals with LFS, whose own health status and that of family members can change rapidly, this needs to be accounted for in recruitment.

### 4.3 Strengths and limitations

This study represents the first German online psychosocial intervention tailored to individuals with LFS, offering unique insights into the intervention needs of this rare and understudied cohort. However, several limitations are seen in the study results. First, in addition to the very small sample size, the findings of this study are likely influenced by selection bias. Second, although questions on current motivation and reasons for dropout were included in each module, no specific reasons were reported. As participants who discontinued did not complete subsequent motivation assessments, these ratings are biased. Additional methods to assess motivation to complete and reasons for drop out would be beneficial in future trials. Third, this study is not able to provide data for all participants due to missing responses. Missing data appeared to result from skipped items, suggesting that questionnaire or intervention length may have been too burdensome. Practicality may also have been impaired by the redirection of participants from OnLiFe to an external survey platform; a more integrated interface could help minimize data loss. Finally, LFS-related events (e.g., interim illness in a family member, surgery) should be documented in future intervention studies to improve understanding of possible reasons for program discontinuation.

## 5 Conclusion

Adults with LFS are at risk for high psychosocial burden due to their rare condition and highly elevated cancer risk. Interventions specifically tailored to this group are scarcely available. This study reports the initial development of a psychosocial self-management program for this population. The results of this study indicate that there is only a small subset of individuals who show interest in the program and complete it, with mixed outcomes regarding acceptability and practicality. The results suggest that a shorter, individually tailored mindfulness-based intervention could be more beneficial for this group; however, further investigation is required. Regarding the actual implementation and further evaluation of OnLiFe, adaptations are required, particularly in relation to content, scope, and design. Open questions remain about the optimal ways to support this specific group.

## Data Availability

The raw data supporting the conclusions of this article will be made available by the authors, without undue reservation.
