# Supplementary material for: A brief feasibility report on an online psychosocial support intervention for adults with Li–Fraumeni syndrome
Source: Front Psychol. 2026 Feb 16;16:1688050. doi: 10.3389/fpsyg.2025.1688050 (PMC12951781; doi:10.3389/fpsyg.2025.1688050)
Supplement: Supplementary file 1 [file Supplementary_file_1.docx]

Supplementary Material

Supplement A: The Hexaflex model of Acceptance and Commitement Therapy, Reprinted with permission from Harris, R. (2009). ACT made simple. An Easy-to-Read Primer on Acceptance and Commitement Therapy. Figure 1, page 10.


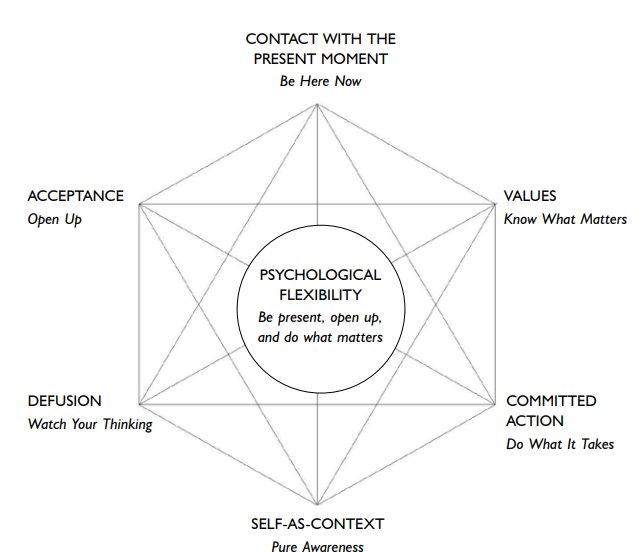


Supplement B: Category system for qualitative feedback on the OnLiFe intervention.

| Main category | Sub-category | Category description | Example |
| --- | --- | --- | --- |
| Positive feedback | Mindfulness exercises | Positive feedback on the (audio-guided) mindfulness exercises | *‘Calming down’ is the goal I want to achieve. This module helped me with that* |
|  | Other exercises | Positive feedback on the non-audio-based exercises, e.g. resources, cognitive restructuring, defusion | *I liked the ‘if then’-exercise. “If I get sick, what could happen then” - that thinking to the end.* |
|  | Psycho-education | Positive feedback on psychoeducational content | *I found the biological explanations of ‘fear’ very helpful.* |
|  | Videos | Positive feedback on the integrated video sequences | *The videos as well ,I thought they were good.* |
|  | General | Positive feedback that is not specified in more detail | *Overall, I had the feeling that I was already on the right track in this area. It was a positive experience with this module.* |
| Negative feedback | Time | Difficulties regarding the time frame (e.g. time pressure) | *I’m not cancelling, but I'm stressed about time. I find it difficult to find the peace and quiet to listen to everything in peace and think it through. Now I've had half an hour and I think it's taken longer. I have to get going IMMEDIATELY.* |
|  | Technology | Difficulties with the technical implementation of the intervention | *Often type on the wrong areas until I find the link for videos or something.* |
|  | Content | Difficulties with the content of the intervention | *It was extremely difficult for me to find examples. I think it's very abstract and complicated.* |
| Change requests | Exercises | Change requests concerning exercises | *I would have liked more exercises.* |
|  | Design of content | Change requests concerning the basic design of the intervention (e.g. level of support) and its content (e.g. type and scope of information provided) | *I would find it more pleasant overall if you could choose a topic. This ‘step-by-step’ approach is tiring.* |
